# The Influence of Triclosan on the Thyroid Hormone System in Humans - A Systematic Review

**DOI:** 10.3389/fendo.2022.883827

**Published:** 2022-06-02

**Authors:** Mai Homburg, Åse Krogh Rasmussen, Louise Ramhøj, Ulla Feldt-Rasmussen

**Affiliations:** ^1^ Department of Medical Endocrinology and Metabolism, Copenhagen University Hospital, Copenhagen, Denmark; ^2^ National Food Institute, Technical University of Denmark, Kgs. Lyngby, Denmark; ^3^ Department of Clinical Medicine, Faculty of Health and Medical Sciences, University of Copenhagen, Copenhagen, Denmark

**Keywords:** triclosan, thyroid, endocrine disrupting chemicals, health, thyroid toxicity, environment

## Abstract

**Objectives:**

Triclosan is an antibacterial agent suspected to disrupt the endocrine system. The aim of this study was to investigate the influence of triclosan on the human thyroid system through a systematic literature review of human studies.

**Methods:**

Eligibility criteria and method of analysis were registered at Prospero (registration number: CRD42019120984) before a systematic search was conducted in Pubmed and Embase in October 2020. Seventeen articles were found eligible for inclusion. Thirteen studies were observational, while four had a triclosan intervention. Participants consisted of pregnant women in eight studies, of men and non-pregnant women in seven studies and of chord samples/newborns/children/adolescents in six studies. The outcomes were peripheral thyroid hormones and thyroid-stimulating hormone (TSH) in blood samples.

**Results:**

Several studies found a negative association between triclosan and triiodothyronine and thyroxine, and a positive association with TSH; however, the opposite associations or no associations were also found. In general, the studies had limited measurement timepoints of thyroid outcomes, and the interventional studies used low concentrations of triclosan. Thus, study design limitations influence the quality of the dataset and it is not yet possible to conclude whether triclosan at current human exposure levels adversely affects the thyroid hormone system.

**Conclusions:**

Further larger studies with more continuity and more elaborate outcome measurements of thyroid function are needed to clarify whether triclosan, at current exposure levels, affects the human thyroid hormone system.

**Systematic Review Registration:**

http://www.crd.york.ac.uk/PROSPERO/display_record.asp?ID=CRD42019120984, identifier PROSPERO (CRD42019120984).

## 1 Introduction

**Figure 1:**
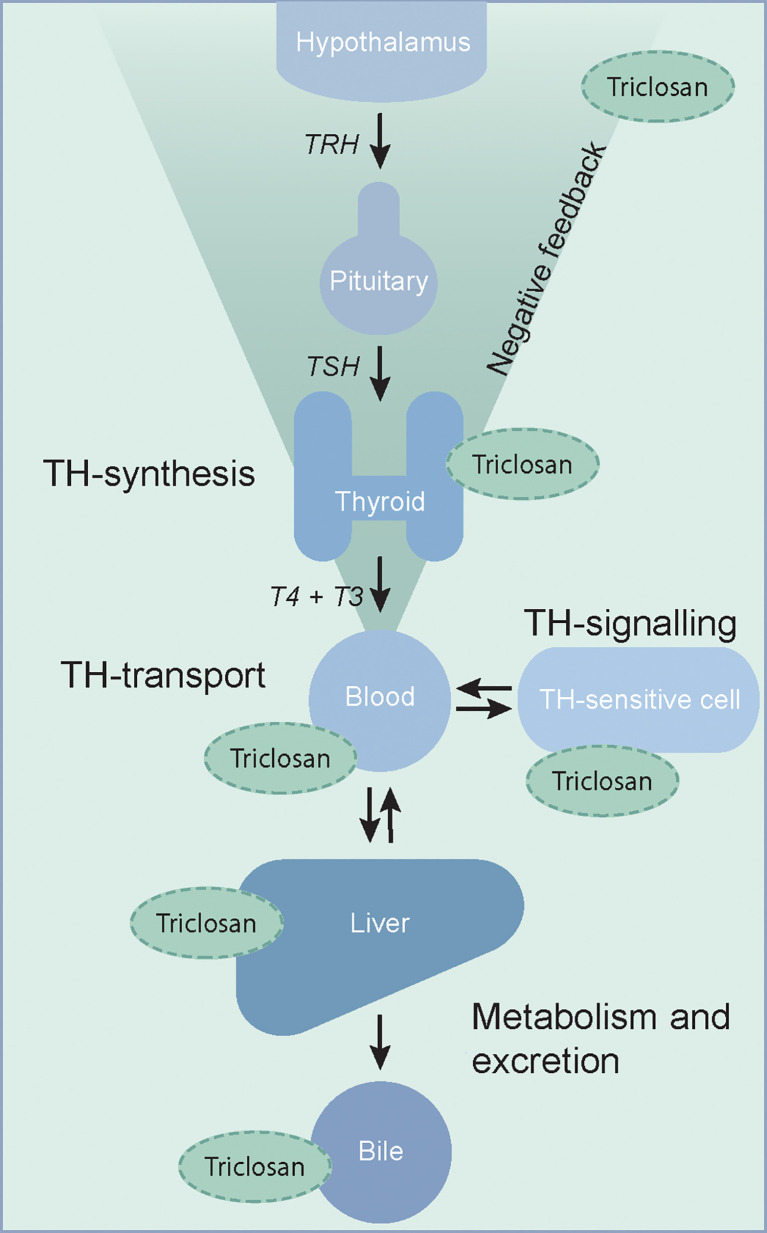
The thyroid hormone system is a complex endocrine system with many potential targets of thyroid hormone system disrupting chemicals. Putative sites of interference by triclosan, as discussed in this review, are indicated. TH, thyroid hormone; T4, thyroxine; T3, 3,3´,5-tri-iodothyronine; TSH, thyroid stimulating hormone; TRH, thyrotropin releasing hormone.

A large number of environmental endocrine disrupting chemicals can affect the thyroid hormone system (1–3). Chemicals can reduce circulating levels of thyroid hormones by a number of mechanisms, including inhibition of iodide uptake and thyroid hormone synthesis in the thyroid gland. However, these are not the only mechanisms of thyroid hormone system disruption and an increasing number of chemicals have been shown to interfere with the thyroid hormone receptor, enzymes or with serum distribution proteins and transporters that play important roles in mediating thyroid hormone action (1–3) (**Figure 1**).

Research endeavours have been intensive within the area and the importance of an optimal thyroid function throughout life for development and maintenance of normal metabolic functions as well as neurological and brain development both in foetal life, and later, has been acknowledged. Nevertheless, the field is still lacking sufficient understanding of the effects on the endocrine systems from the numerous chemicals utilized in everyday household and cosmetic products.

Triclosan (5-Chloro-2-(2,4-dichlorophenoxy)phenol) has been under investigation for its possible endocrine disrupting effects in humans through the last decades (4, 5). Triclosan is an antibacterial agent used in industrial, personal and household products, such as deodorants and toothpaste (4) and it can be detected in the urine of 97.1% of young Danish men (6), although with a decreasing tendency over 8 years (7). This decreasing tendency was in agreement with results from The National Health and Nutrition Examination Survey (NHANES) of nationally representative sample of about 5,000 persons each year from different places of United States of America. However, in the NHANES study covering data from 2013-14 (8) the concentration was about three to ten times higher in young American compared to the young Danish men.

**Figure 2:**
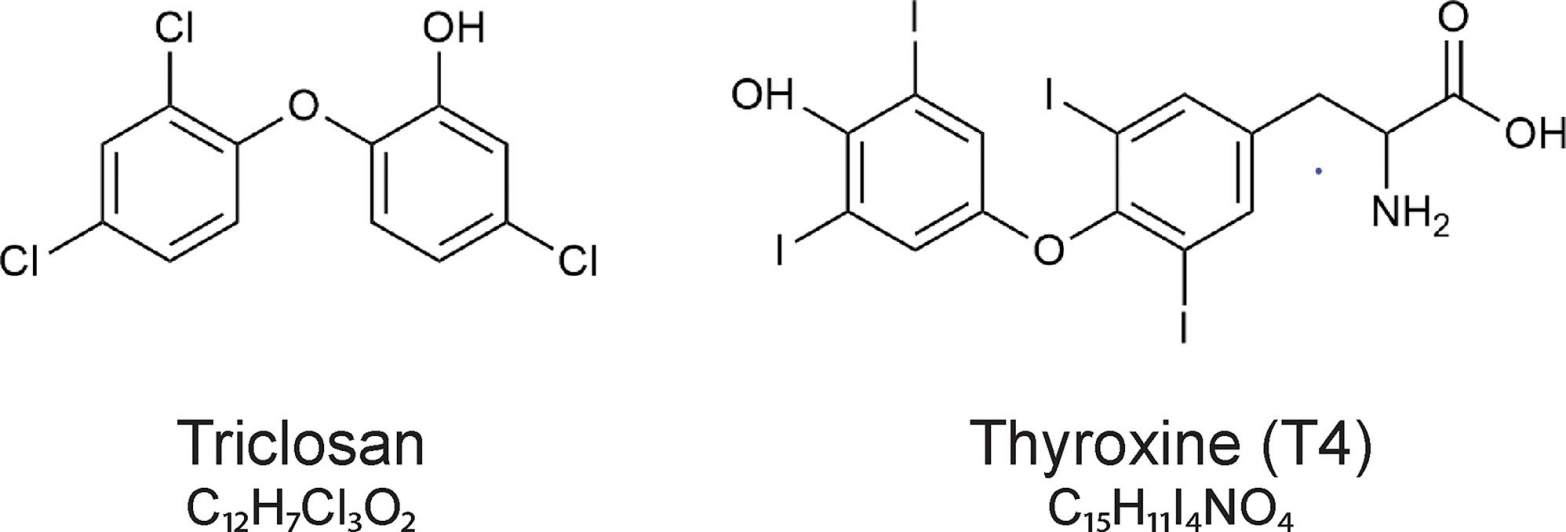
The chemical structures of triclosan (5-chloro-2-(2,4-dichlorophenoxy)phenol)) and the thyroid hormone thyroxine (T4).

Structurally, triclosan resembles thyroxine (T4), (**Figure 2**) and it can disrupt the thyroid hormone system (9–12). Thus, in rodents, triclosan consistently reduces serum T4 concentrations, probably by increasing hepatic catabolism of thyroid hormones (9, 13, 14).

In the European Union triclosan is registered under the REACH (Registration, Evaluation, Authorization and Restriction of Chemical substances) regulation and it is under evaluation as an endocrine disruptor (15, 16). Triclosan is currently not being manufactured in or imported to the European Economic Area (16) but exposure appears to be continuing with triclosan still being found in the urine of young Danish men (7). The aim of this study was to investigate the effect of triclosan on the human thyroid hormone system through a systematic literature review of human studies.

## 2 Methods

As guidance in the process of this systematic review, we used the PRISMA statement for reporting systematic reviews (17) and Cochrane Handbook for systematic reviews.

### 2.1 Study and Report Criteria

The method of analysis and criteria have been specified in advance and registered in two protocols concerning respectively human and animal studies at Prospero (registration number: CRD42019120984). Here we report the results from the protocol concerning the human studies. All human studies concerning the effect of triclosan on thyroid function, thyroid growth and thyroid cell morphology directly were included in the search. The studies had to be in English and with original data. No restrictions were imposed concerning publication date, type of participants or type of outcome measures, except that details of the method used must be reported. The only restriction toward type of intervention was exclusion in case of deliberate exposure to other potential endocrine disruptors in combination with triclosan (supplementary material).

### 2.2 Database Search and Study Selection

Studies were identified by systematic searches on Pubmed (Medline/US National Library of Medicine), 1946 to present) and Embase (1974 to present, provider: Ovid). The searches were done in October 2020.

Search terms were selected through MeSH and “search-tools” search in PubMed and Embase, respectively. The full search strategy can be seen in the supplementary material.

### 2.3 Data Extraction and Analysis

One author (MH) screened titles and abstracts for reports that matched eligibility criteria. If doubt of inclusion, full text was read, and a second author (ÅKR or UFR) was consulted. A data-extraction sheet was developed by MH and data was collected regarding study design, participant characteristics, tissue studied and thyroid endpoints, primary outcome of the study, timing of outcome measurement and method used, other relevant outcomes, confounders and information to assess risk of bias. Further data for interventional studies was collected regarding intervention groups, route of administration, dose and frequency of exposure.

If the authors of more studies were the same and study participants were similar, the study characteristics and outcomes were compared to avoid replicates.

The risk of bias in the included studies was assessed by guidance of the Cochrane Collaboration’s tool for assessing risk of bias.

## 3 Results

### 3.1 Study Selection

**Figure 3:**
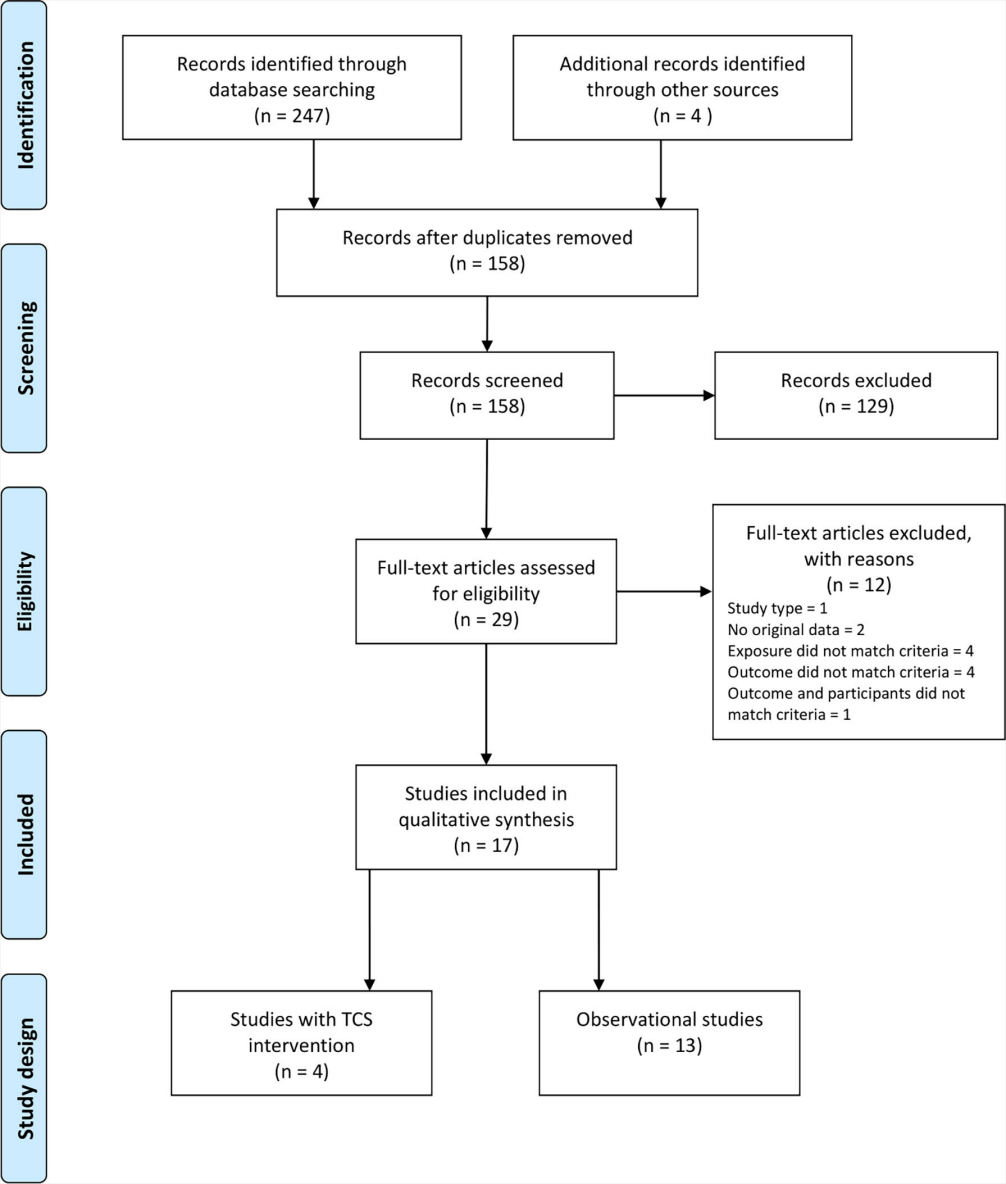
PRISMA diagram of selection of eligible studies (4 studies of triclosan intervention and 13 observational studies) included in the search for studies of triclosan exposure and human thyroid function.

When searching the two databases 247 records were identified, and 154 remained after removal of duplicates. Additional four articles were found through other sources. Screening of the 158 titles and abstracts led to exclusion of 129 reports. The remaining 29 reports were read in full text and assessed for eligibility. Twelve reports did not match eligibility criteria and were excluded. Finally, a total of 17 reports met the criteria and were included in the review (**Figure 3**).

### 3.2 Study Characteristics

The study characteristics of the 17 included human studies are summarised in **Table 1**. Thirteen studies were observational, while four had a triclosan intervention.

**Table 1:** Characteristics of the articles (four studies with triclosan intervention and 13 observational studies) obtained from searches at PubMED (1946 to Oct 2020) and Embase (1974 to Oct 2020) of human studies concerning the effect of triclosan on the thyroid hormone system.

Source	Methods:	Participants + material:	Intervention and/or duration of study:	Outcomes:
**Studies with TCS-intervention**				
Ley et al., 2017 (18)	Nested randomized clinical trial. Non-blinded.	70 pregnant women/new mothers (TCS-group: 31, non-TCS group: 39): Thyroid: Serum. TCS: Urine	Dermal, orally (Toothpaste, soap, etc.) Exposure: app. 23,7-25,3 weeks	tT3, tT4, fT4, TSH. Intervention vs. control group and high vs. low urinary TCS. Three time points.
Poole et al., 2016 (19)	Randomized cross-over control study. Blinded	13 adults: Thyroid: Serum TCS: Urine	Orally, dermally. (Toothpaste, soap, etc.) Exposure: 4 months	T4, TSH. Two groups with crossover. TCS phase vs. no TCS vs. baseline
Palmer et al., 2011 (20)	Substudy of a randomized, clinical trial. Blinded	132 adults (TCS-group: 64, Placebo: 68): Thyroid: Serum	Orally (0.3% TCS containing toothpaste) Exposure: 4-5 years	fT3, fT4, TSH, TgAb, TPOAb. Intervention vs. control group. Two time points.
Allmyr et al., 2009 (21)	Pre-post clinical trial. Non-blinded.	12 adults: Thyroid and TCS: Plasma	Orally (toothpaste) Exposure: 2 weeks	fT3, fT4, TSH, 4beta-hydroxycholesterol. Before vs. after exposure
**Observational studies**				
Almstrup et al., 2020 (22)	Nested longitudinal subcohort of a cross-sectional study	51 children (pre- and postpubertal): TCS: Urine x2-3 TH: Serum x2	Samples from pre- and postpubertal children	DNA-methylation of TRIP6
Guo et al., 2020 (23)	Cross-sectional study	386 mother singleton-pairs. Pregnant women: TCS: urine x1 Chord sample: TH: serum x1	Single sample	tT3, tT4, fT3, fT4, TSH, TPOAb, TGAb
Ha et al., 2019 (24)	Cross-sectional study	5990 adults: TCS: Urine x1 TH: Serum x1	Single sample	tT3, tT4, TSH
Derakhshan et al., 2019 (25)	Cross-sectional study	1996 pregnant women: TCS: Urine x1 TH: Serum x1	Single sample	tT3, tT4, fT3, fT4, TSH, TPOAb, TgAb
Aker et al., 2019 (26)	Cohort study with repeated measures	602 pregnant women: TCS: urine x 3 TH: serum x 2	Samples from a period of 4-12 weeks	tT3, tT4, fT4, TSH, T3/T4ratio
Skarha et al., 2019 (27)	Cross-sectional study	317 women TCS: Urine x1 TH: Serum x1	Single sample	tT3, tT4, fT3, fT4, TSH, TPOAb, TgAb
Berger et al., 2018 (28)	Cohort study with repeated measures	338 pregnant women: TCS: Urine x2 (a mean was used) TH: Serum x1 364 newborns (heel stick): TH: Serum x1	Urine samples from a period of app. 13 weeks. Single blood sample from each participant.	Women: fT4, tT4, TSH Neonates: TSH
Aker et al., 2018 (29)	Nested case-control study with repeated measures	439 pregnant women (116 preterm birth cases and 323 term births as controls) TCS: Urine x4 TH: Plasma x4	Samples from a period of app. 25 weeks	tT3, tT4, fT4 and TSH
Braun et al., 2018 (30)	Cohort study with repeated measures	350 mother-child pairs Pregnant women: TCS: Urine x3 TH: Serum x1 Chord sample: TH: Serum x1 Children 3y: TCS: Urine x3 TH: Serum x1	Urine samples from a period of app. 24 weeks for pregnant women and two years for children	tT3, tT4, fT3, fT4, TSH, TPOAb, TgAb
Wang et al., 2017 (31)	Cohort study with repeated measures	398 mother-infant pairs Pregnant women: TCS: Urine x1 TH: Serum x1 Chord sample: TH: Serum x1	Single sample from each participant	Maternal: fT4, TSH and TPOAb Umbilical cord: fT3, fT4, TSH, TPOAb
Aker et al., 2016 (32)	Cohort study with repeated measures	106 pregnant women. TCS: Urine x2 TH: Serum x2	Samples from a period of 4-12 weeks	fT3, fT4, TSH
Geens et al., 2015 (33)	Cohort study with repeated measures and case-control. Indirect intervention	194 adults (151 obese and 43 lean) Obese: TCS: Urine x4 TH: Serum x4 Lean: TCS: Urine x1 TH: Serum x1	6 months	fT4, TSH
Koeppe et al., 2013 (34)	Cross-sectional study	1831 persons ≥12 years: TCS: Urine x1 TH: Serum x1	Single sample	tT3, tT4, fT3, fT4, thyroglobulin, TSH

TCS, triclosan; tT3, total triiodothyronine; fT3, free triiodothyronine; tT4, total thyroxine; fT4, free thyroxine; TSH, thyroid-stimulating hormone; TPOAb, antithyroid peroxidase antibodies; TgAb, antithyroglobulin antibodies; TRIP6, thyroid hormone receptor interactor 6 gene.

All 17 human studies measured thyroid function outcomes in blood samples and when the triclosan concentration was assessed, it was measured in urine samples. Eight of the studies used serum from pregnant women (18, 25, 26, 28–32). Three studies used chord serum samples (23, 30, 31) and four used serum from newborns/children/adolescents (22, 28, 30, 34). Seven studies investigated men and non-pregnant women (19–21, 24, 27, 33, 34).

One interventional study was randomized (n=70) (18), two were randomized and blinded trials (n=132, n=13) (19, 20), while one study compared the outcome before and after the triclosan intervention (n=12) (21).

The triclosan interventions consisted of household and personal care products containing triclosan in two of the studies (18, 19) and only triclosan containing toothpaste in the other two (20, 21). Three of the interventional studies measured the triclosan concentration in blood or urine after exposure, but only one assessed the association between the actual measured triclosan concentration and thyroid endpoints (18). The exposure time varied between two weeks and five years.

### 3.3 Risk of Bias Within Studies

The four intervention studies were assessed for risk of bias by using a modified version of the Cochrane Collaboration’s “Risk of Bias” tool. Because the majority of included studies were cross sectional, we chose to use JBI Critical appraisal tool specifically for cross sectional studies (35, 36) (**Supplementary Material, Tables 3, 4**).

In general, the observational studies informed about eligibility criteria, study population, confounding factors, exposure and outcome measurements. Most of the interventional studies took risk of bias into account in their study design. However, unclear information about randomization method, blinding and handling of incomplete outcome data prevented thorough risk of bias assessment.

### 3.4 Results of Individual Studies

The results of the included studies are summarised in **Tables 2** and **3**.

**Table 2:** Summary of findings of associations between triclosan concentrations and thyroid hormone system variables in observational studies of adult humans.

Study	T3(fT3 and/or tT3)	T4(fT4 and/or tT4)	TSH	TPOAb	TgAb	Other outcomes
*Subject for thyroid outcome analysis*						
**Almstrup et al. 2020 (****22****)**						
* Pre- and postpubertal Children*						(TRIP6 promoter methylation)→
**Guo et al. 2020 (****23****)**						
* Neonates*	→	→	→	→	**-**	
**Ha et al. 2019 (****24****)**						
* Females*	→	→	→			
* Males*	→	→	→			
**Derakhshan et al. 2019 (****25****)**						
* Pregnant women*	→	→	→			
**Aker et al. 2019 (****26**)						
* Pregnant women*	→	→	→			
**Skarha et al. 2019 (****27**)						
* Women*	↓→	→	→	↓	→	
**Berger et al. 2018 (****28**)						
* Pregnant women*		→	→			
* Neonates*			→			
**Aker et al. 2018 (****29**)						
* Pregnant women*	↓	→	↑			
**Braun et al. 2018 (****30**)						
* Pregnant women*	→	→	→			
* Neonates*	→	→	→	→	**-**	
3y old children	→	↑→^1^	↑^2^	↑→^3^	**-**	
**Wang et al. 2017 (****31**)						
* Pregnant women*		↓	→	→		
* Neonates*	↓	→	→	→		
**Aker et al 2016 (****32**)						
* Pregnant women*	→	→	→			
**Geens et al. 2015 (****33**)						
Obese adults, all individuals and in female		↓	→			
Obese adults, male		→	→			
Weightloss, 3 months, all individuals and in male		→	→			
Weightloss, 3 months, female		↓	→			
Weightloss, 6 months (all)		→	→			
Lean adults (all)		→	→			
**Koeppe et al. 2013 (****34**)						
12-19y all	→↑	→	→			(Tg) →
12-19y female, male	→	→	→			(Tg) →
>19 y all, female, male	→	→	→			(Tg) →

↑: positive association, ↓: negative association, →: no association.

Crude/unadjusted models if possible.

^1^The increase in T4 is when the urinary triclosan concentration is from the child.

^2^The increase in TSH is when the urinary triclosan concentration is from the pregnant mother.

^3^The increase in TPOAb is when the urinary triclosan concentration is from the one-year old child.

tT3, total triiodothyronine; fT3, free triiodothyronine; tT4, total thyroxine; fT4, free thyroxine; TSH, thyroid-stimulating hormone; TPOAb, antithyroid peroxidase antibodies; TgAb, antithyroglobulin antibodies; TRIP6, thyroid hormone receptor interactor 6 gene; Tg, thyroglobulin.

Neonates, children, pregnant women and adults are marked in different colours.

**Table 3:** Summary of findings of associations between triclosan concentrations and thyroid gland variables in interventional studies of human adults.

Study	T3 (fT3 and/or tT3)	T4 (fT4 and/or tT4)	TSH	TPOAb	TgAb	Other outcomes
*Subject for thyroid outcome analysis*						
**Ley et al. 2017 (****18**)						
* Pregnant women/mothers*	→	→	→			
**Poole et al. 2016 (****19**)						
* Adults*		-	→			
**Palmer et al. 2011 (****20**)						
* Adults*	→	→^1^	→	→	→	
**Allmyr et al. 2009 (****21**)						
* Adults*	→	→	→			→(4β-hydroxycholesterol)

↑: positive association, ↓: negative association, →: no association.

^1^ At 5 years follow-up T4 was significantly lower in the placebo group than in the Triclosan group.

tT3, total triiodothyronine; fT3, free triiodothyronine; tT4, total thyroxine; fT4, free thyroxine; TSH, thyroid-stimulating hormone; TPOAb, antithyroid peroxidase antibodies; TgAb, antithyroglobulin antibodies; TRIP6, thyroid hormone receptor interactor 6 gene; Tg; thyroglobulin.

Pregnant women and adults are marked in different colours.

#### 3.4.1 Results of Individual Studies – Interventional (n=4)

Four studies investigated the effect of triclosan on thyroid hormones by interventional experiments and none found a significant effect (18, 19, 21), except for one study observing a decrease in free T4 (fT4) in the placebo group (20).

The two studies who exposed participants through triclosan-containing household and/or care products observed a significant increase in urinary triclosan after the intervention (18, 19). However, none of the two studies observed a significant association between the intervention arm and T4 and thyroid-stimulating hormone (TSH), respectively after four months, nor of total triiodothyronine (T3), total T4, fT4 and TSH after approximately 23 weeks of exposure. There was no association between urinary triclosan concentration and serum thyroid hormones (18).

Two studies investigated the effects of triclosan-containing toothpaste (20, 21). Palmer and co-workers did not find an association between triclosan and free T3 (fT3), TSH, antithyroglobulin antibodies (TgAb) nor antithyroid peroxidase antibodies (TPOAb) when comparing the intervention and control group after 4 years of exposure. However, fT4 was significantly decreased after 4 years use of placebo, while the triclosan-exposed participants had an unchanged concentration (20). The other study, on the use of toothpaste, did not find an association between triclosan and fT3, fT4, TSH nor of 4beta-hydroxycholesterol, respectively, before and after 2 weeks of exposure, although the plasma triclosan concentration was significantly increased (21).

#### 3.4.2 Results of Individual Studies – Observational n=13

Ten studies assessed the associations between urinary triclosan concentrations and serum T3 while 12 studies measured T4. The associations to TSH were investigated in 12 studies while that on TPOAb and TgAb in 5 and 4 studies, respectively.

##### 3.4.2.1 Triclosan and T3

Ten studies assessed the associations of triclosan to the concentration of T3 (23–27, 29–32, 34). Four of them found a significant association between triclosan and T3 - three found triclosan associated with a decrease in T3, while one found an increase. The decrease in T3 was seen in studies of 317 women with no history of hypo- or hyperthyroidism seeking medically assisted reproductive treatment, 439 pregnant women and 398 mother-infant pairs (27, 29, 31), while the increase was seen in adolescents (34).

Several models were adjusted for demographic factors and other confounders; however, it did not lead to any major changes in the results or the conclusions.

##### 3.4.2.2 Triclosan and T4

Nine of the twelve studies on the associations between triclosan and T4 did not find a significant effect (23–29, 32, 34), while three did (30, 31, 33). Two of these three studies found an association between triclosan and a decrease in T4, while the last found triclosan related to an increase (30).

One study followed pregnant women and their children until the age of three and found a positive association between childhood urinary triclosan and T4 concentrations in 3-year olds, however similar associations were not found in pregnant women or neonates (30). Another study of pregnant women found that triclosan was associated with a decrease in T4, while no association was found between maternal urinary triclosan and T4 in cord serum samples (31). A study of obese persons undergoing a weight loss found a negative association between fT4 and urinary triclosan before the weight loss. After three months of weight loss this association was only seen in females (33).

Several models were further adjusted for demographic factors and other confounders, but no substantial changes were seen, apart from one study of pregnant women describing a decrease in T4 after adjustments for maternal age, education, country of birth, poverty index at baseline and similarly when further adjusted for benzophenone-3, triclosan and the sum of dialkyl phosphate metabolites (data not shown) (28).

##### 3.4.2.3 Triclosan and TSH

Moving to the pituitary response to peripheral thyroid hormones, ten of 12 studies did not find an association between triclosan and TSH (23–28, 31–34) while two studies demonstrated an increase in TSH with increasing triclosan concentration in the urine (29, 30). In one of the studies including pregnant women, this increase disappeared when the models were stratified by gestational age (29). The other study followed pregnant women and their children until the age of three. They found a positive association between gestational urinary triclosan and serum TSH in the children at age three, but not in the pregnant women nor in the neonates (30). The association in children was not significant when the model was adjusted for demographic factors (data not shown), however, no changes were observed in the remaining studies after adjustment for gestational age, body mass index etc.

##### 3.4.2.4 Triclosan and Thyroid Autoantibodies (TPOAb, TgAb)

Five studies measured thyroid autoantibodies (25, 27, 30, 31, 37). Two of these found a significant association between TPOAb and triclosan (27, 30), while one assessed TPOAb and TgAb as confounders in a cross sectional study of 1996 pregnant women (25). They described that the association of triclosan with TSH or T4 did not differ according to TPOAb status (p>0.05). No calculations were mentioned for TgAb.

A cross-sectional study of 317 women described a negative association between TPOAb and urinary triclosan (27). Another study followed pregnant women and their children until the age of three (30). Here, 328% higher (95% CI: 18, 1457) TPOAb levels in three years olds were positively associated with each 10-fold urinary triclosan level at the age of one. No association was found with gestational or other childhood triclosan concentrations, nor with TPOAb levels at delivery (30).

##### 3.4.2.5 Triclosan and Other Outcomes

Serum thyroglobulin concentrations were measured in a single study of 1831 men and women at the age of 12 and older. Here, there were no statistically significant associations with triclosan in any analyses (34). Another study investigated the association between triclosan and the promoter methylation of the thyroid hormone receptor interactor 6 gene (*TRIP6*) in 51 pubertal children and did not find any association (22).

## 4 Discussion

This review explores the potential effects of triclosan on thyroid parameters in humans. The outcomes investigated in nearly all studies were the pituitary hormone TSH and the thyroid hormones, T4 and T3. T4 is produced and released from the thyroid gland in larger quantities than the more active T3. Most of the T4 is deiodinated to T3 or the inactive reverse T3 in peripheral tissue cells, where T3 can bind to, and activate nuclear receptors. T4 is thus considered a prohormone. Measurement of the hormones in serum can be as total T4 and T3 measurements: the free hormones plus the hormones bound to the binding proteins thyroxine-binding globulin, albumin and transthyretin are measured. Alternatively, measurements can be made as an estimate of the free hormone concentrations (fT4 and fT3), which comprises a very small fraction: of the hormones in the circulation. These small amounts result in a high variability in measurements partly due to the low concentrations, partly to the protein binding and risk of disturbance of equilibrium by physiological, pathophysiological and pharmacological interferences (38). It has not been established, if triclosan can disturb this equilibrium and influence measurements of free thyroid hormone in urine and plasma although it is possible due to the ability of triclosan to bind the serum distribution protein transthyretin (39–43).

The 13 observational studies were all judged to have a low risk of bias (**Supplementary Material, Table 3**). Of the 4 intervention studies, two of them lacked blinding (18, 21) and in two it was impossible to judge attrition bias because of missing information on incomplete outcome data (18, 19). Despite the more positive judgement of risk of bias in the observational than in the interventional studies, observational studies should nevertheless in general be evaluated more critically than randomized controlled clinical trials.

Based on the 17 included studies, it is not possible to determine the potential effect of triclosan on the human thyroid system at current exposure levels. Although several studies found a negative association between triclosan and T3 and T4, and a positive association with TSH; the opposite associations as well as no association in the majority of the studies were found. The results are thus still ambiguous. Most of the included studies investigated women and children, both of whom are more prone to thyroid diseases than men. During development adequate and timely supplies of the thyroid hormones are essential for fetal and childhood development, particularly for the nervous system and cognitive function, but also for growth (44). However, it is difficult to interpret thyroid status in pregnant women due to pronounced physiological changes during pregnancy, including a necessary physiological increase in thyroid hormone binding proteins, which by definition changes measurements and conclusions for both total and free hormones (45). The final interpretation of thyroid hormone concentrations are therefore in each case best based on a combination of TSH, total T4 and T3 and fT4 and fT3 estimates as well as thyroid binding protein concentrations (46).

One of the limitations of this study was that the majority of included studies were cross sectional or repeated measures studies with two to four samples. This is a result of the strict inclusion criteria and partly a consequence of the questions addressed in this review. Ethically, it is not possible to administer high amounts of triclosan in a randomized clinical trial. Furthermore, a satisfactory exclusion of other confounders in the form of potential presence of other endocrine disrupting chemicals (EDCs) is very difficult without a high degree of isolation from everyday products. This makes good randomized clinical trials difficult to execute. Based on observational studies we cannot establish causal relationships between triclosan exposure and measured outcome, and exposure to other substances as well as other confounders may influence the results.

Four of the included studies were clinical trials exposing participants to triclosan *via* the daily use of personal care products such as toothpaste and soap. The concentration of triclosan in the intervention products was low and exposure reflected a realistic triclosan exposure from the use of a single triclosan-containing product, but this compromised distinction from other EDCs and exposure to triclosan from multiple sources. Furthermore, the numbers of participants were small. The strength of evidence in the interventional studies is therefore not clearly higher than in the cross-sectional studies.

To use a single or few blood and urine samples to assess the association between triclosan and the thyroid system, does not elaborate the possible effects of triclosan adequately. Both because of the possible changes in the association over time and because of the numerous interactions in the endocrine system and with other EDCs. Investigations of limited parts of the system are consequently uncertain (**Figure 1**). The applicability of the studies is especially debatable when regarding the eight studies of pregnant women, since the thyroid hormone homeostasis is particularly dynamic during pregnancy. In the beginning of a pregnancy, the amount of thyroxine-binding globulin rises drastically because of estrogen stimulation of the hepatic production (47). At the same time, the thyroid hormone requirement is increasing as a consequence of fetal thyroid hormone consumption, plasma volume expansion, thyroid hormone metabolism, and increase in renal clearance of iodide, and finally conversion of T4 to reverse T3 in the placenta by deiodinase 3 also increases (47, 48). This requires the thyroid gland to increase thyroid hormone synthesis, although changes in the measured serum hormone concentrations are rather small. Still, this renders the pregnant thyroid hormone system vulnerable to exogenous stressors. Total T3 and T4 reach a plateau around gestational week 20, but until this point, T4 and thyroxine-binding globulin levels are constantly changing and exhibit wide individual variation (49, 50). Therefore, the associations may vary considerably according to the measurement time point and it is possible that the effect of triclosan also varies over the course of pregnancy (44, 51).

One study included measurements throughout pregnancy and collected blood and urine in all three trimesters, this study found no associations between triclosan and fT4, total T4, total T3 or TSH at any time point (29). However, most of the included observational studies of pregnant women had only one or two measurement timepoints. Thus, variations between individuals and their pregnancy timing can reduce the statistical power of the studies and the differences in study designs impairs comparisons between the studies. Although the thyroid hormone balance is more stable in non-pregnant individuals the limited measurement timepoints are also in this respect critical for the power of the studies.

Aker and co-workers suggested in their study from 2019 (26) that the decreased total T3 could be a result of the structural similarity between triclosan and T3/T4. This corresponds with the speculations of Wang et al, that triclosan acts as negative feedback on the hypothalamic-pituitary-thyroid axis with inhibition of TSH secretion as a result (31). This could occur by imitating the effect of T3/T4 in the negative feedback loop. The thyrotrophs monitor the intracellular T3 concentration, where already formed T3 enters from plasma and deiodinase type II transforms T4 to T3. T3 subsequently decreases the number of thyrotropin releasing hormone (TRH) receptors on the surface of the thyrotrophs and also inhibits the synthesis of TSH (52). Another explanation could be through inhibition of the TRH-receptor or the successive phospholipase c pathway which upon stimulation increases synthesis and release of effect of TSH (52). Wang et al. found an association between a reduction of serum TSH and the medium tertile of urinary triclosan concentrations (31), which could be in keeping with above theories.

Conversely, Aker et al. demonstrated an increase in TSH in their study from 2018 (29) and argued that if triclosan was responsible for the decrease in T3, then TSH would increase given the negative feedback loop. This consequently implies an assumption that triclosan does not inhibit the TSH release. An alternative explanation could be that triclosan inhibits the effect of TSH effects in the thyrocytes, which would reduce the T3 and T4 synthesis and release, and lead to a rise in TSH. However, many other mechanisms act on the feedback loop, among other somatostatin and dopamine (52).

Most included studies found a decrease in either T3 or T4, however Koeppe et al. (34) and Braun et al. (30) found increases in T3 and T4, respectively. Sulfation is the rate-limiting step for T3 and glucuronidation for T4 in biliary excretion of thyroid hormones (53), and triclosan has been shown to inhibit both enzymes, which could explain the increase (54, 55). Paul and co-workers oppositely found increased T4-glucuronidation, and phase II enzymes responsible for thyroid hormone catabolism in the liver were upregulated in rodents (9).

The mechanisms by which triclosan potentially disrupts the thyroid hormone system has been assessed in numerous *in vitro* studies of human cell lines and cytosol. Butt and coworkers (55) investigated the effect of triclosan on deiodinases from human liver cells (56). Only type 1 of the outer ring-deiodinases is present in the liver, and triclosan inhibited its transformation of T4 to T3. Type 2 deiodinase is present in the CNS, where it transforms T4 to T3 and thereby affects the negative feedback loop of thyroid hormones and TSH. It could be interesting to investigate whether triclosan also inhibits type 2 deiodinase. The inhibition of type 1 deiodinase alone would lead to a decrease in fT3 without affecting the TSH concentration, while a simultaneous inhibition of type 2 deiodinase would lead to a rise in TSH. No effect of triclosan has been observed on the inactivation of T4 to reverse T3 by inner ring deiodination (56).

Triclosan can also inhibit iodotyrosine deiodinase, also known as iodotyrosine dehalogenase 1 or DEHAL1 (57). Iodotyrosine deiodinase action normally results in the release of iodide and tyrosine from the two products 3-iodo-l-tyrosine and 3,5-diiodo-l-tyrosine, which are released along with T3 and T4 during the thyroglobulin proteolysis. This can lead to a lack of adequate iodide retention and a decrease in T3 and T4 (57).

Several studies found that triclosan could bind to transthyretin receptors and were capable of displacing T4 (39–43). It is suggested that this displacement leads to a larger amount of available free T4 for hepatic uptake, conjugation and biliary elimination (43). This mechanism may be more relevant for rodents, since humans have a large reserve of T4 (75%) stably bound to thyroid binding globulin (58), however it is suggested that the disruption of transthyretin binding will lead to a change in the delivery of free T4 to target cells (43). Furthermore, transthyretin is important in humans because of its role in mediating the delivery of T4 to the human foetus’ through the placenta and across the blood brain barrier and in transport of thyroid hormones within the brain (39).

Several experimental animal studies have shown that triclosan reduces the level of T4 in rodents (14), but without an effect on TSH (9, 12, 59). Thus, a concern for potential human health effects of triclosan exposure is warranted, also in the wake of equivocal human evidence. This, in particular because of the challenges in performing epidemiological studies of chemical exposure and the thyroid hormone system and due to the combined human exposure to environmental chemicals.

## 5 Conclusion

This systematic literature review has investigated the potential effects of triclosan on the human thyroid system at current exposure levels. Several studies found a negative association between triclosan and T3 and T4, and a positive association with TSH, however, opposite observations were also seen, and many of the studies did not find any association at all. Thus, our conclusion is that it is still equivocal whether triclosan, at current human exposure levels, affects the human thyroid hormone system. This is in agreement with several earlier published studies in the field. Most of the included studies assessed the thyroid outcome through few blood samples which decreases the applicability of the studies, especially concerning pregnant women who have a particularly dynamic thyroid hormone homeostasis. Since triclosan potentially causes harm to human health, further and more extensive cohort studies with numerous measurement timepoints for the thyroid outcomes are needed, as are studies with designs that take mixture effects of multiple endocrine disrupting chemicals into account.

## Data Availability Statement

The original contributions presented in the study are included in the article/**Supplementary Material**. Further inquiries can be directed to the corresponding author.

## Author Contributions

UF-R, ÅKR, and MH contributed to conception and design of the study. LR was consultant. MH wrote the first draft of the manuscript. UF-R and ÅKR wrote sections of the manuscript. All authors contributed to manuscript revision, and read and approved the submitted version.

## Funding

MH received unrestricted grants from The Novo Nordisk Foundation (grant number: NNF18OC0052576), and ‘Musikforlæggerne Agnes og Knut Mørks Fond’. UF-R’s research salary was sponsored by a donation from The Kirsten and Freddy Johansen’s Foundation.

## Conflict of Interest

The authors declare that the research was conducted in the absence of any commercial or financial relationships that could be construed as a potential conflict of interest.

## Publisher’s Note

All claims expressed in this article are solely those of the authors and do not necessarily represent those of their affiliated organizations, or those of the publisher, the editors and the reviewers. Any product that may be evaluated in this article, or claim that may be made by its manufacturer, is not guaranteed or endorsed by the publisher.
