# Supplementary material for: The Influence of Triclosan on the Thyroid Hormone System in Humans - A Systematic Review
Source: Front Endocrinol (Lausanne). 2022 Jun 2;13:883827. doi: 10.3389/fendo.2022.883827 (PMC9202756; doi:10.3389/fendo.2022.883827)
Supplement: Supplementary file 1 [file DataSheet_1.docx]

Supplementary Material

# Criteria

## Exclusion criteria

1. Other languages than English
2. Publications with only the abstract in English
3. Reviews
4. No original data
5. All other study types than in vivo studies
6. No report of changes in thyroid function, thyroid growth or thyrocyte morphology related to triclosan exposure
7. No report of duration of exposure
8. No report of concentration of triclosan
9. No report of timing of outcome measure
10. No report of details of specific methods used to measure the outcomes
11. No relevant outcomes reported. Indirect effects of the thyroid function as outcome (e.g. neurological function).
12. Studies will be excluded, if the exposure to triclosan deliberately is combined with exposure to other potential endocrine disrupters, since we wish to see the isolated effect of triclosan.

## Inclusion criteria

- Study subjects should be humans
- All sorts of exposure to triclosan In vivo (per oral, per inhalation, dermal, intra dermal, inter peritoneal, intra venous etc.).
- Studies with or without comparators/controls.
- All types of studies with original data will be included.
- Main outcomes: Changes in thyroid function, thyroid growth and thyrocyte morphology related to triclosan exposure must be reported.

# Full search strategy

Pubmed

Start and end date of search: January 2019 until October 2020 (102 hits)

1. Thyroidea

2. "thyroid gland"

3. Thyroids

4. Thyroid

5. glands, thyroid

6. gland, thyroid

7. thyroid gland

8. thyroid glands

9. glandula thyroidea

10. isthmus glandulae thyroidea

11. thyroid gland isthmus

12. thyroid isthmus

13. hypophysis thyroid system

14. thyroid cell

15. thyroid follicle

16. thyroid lobe

17. thyroid parenchyma

18. thyroid function

19. euthyroidism

20. #1 or #2 or #3 or #4 or #5 or #6 or #7 or #8 or #9 or #10 or #11 or #12 or #13 or #14 or #15 or #16 or #17 or #18 or #19

21. ch 3565

22. cloxifenol

23. dp 300

24. dp300

25. methyltriclosan

26. Irgasan-DP300

27. 5-chloro-2- AND (2,4-dichlorophenoxy) AND phenol)

28. Triclosan

29. 2,4,4'-Trichloro-2'-Hydroxydiphenyl Ether

30. 2-Hydroxy-2',4,4'-trichlorodiphenyl Ether

31. irgasan DP 300

32. irgasan DP300

33. DP300

34. Irgasan

35. #21 or #22 or #23 or #24 or #25 or #26 or #27 or #28 or #29 or #30 or #31 or #32 or #33 or #34

36. #20 AND #35

Embase, ovid:

Start and end date of search: January 2019 until October 2020 (145 hits)

1. glandula thyroidea.mp. or thyroid gland/

2. isthmus glandulae thyroidea.mp. or thyroid gland/

3. thyroid.mp. or thyroid gland/

4. thyroid gland isthmus.mp. or thyroid gland/

5. thyroid isthmus.mp. or thyroid gland/

6. (glandula thyroidea or isthmus glandulae thyroidea or thyroid or thyroid gland isthmus or thyroid isthmus).af.

7. exp thyroid gland/ or exp hypophysis thyroid system/ or exp thyroid cell/ or exp thyroid follicle/ or exp thyroid lobe/ or exp thyroid parenchyma/

8. exp thyroid function/ or exp euthyroidism/

9. (thyroidea or thyroids or thyroid or gland, thyroid or glands, thyroid or thyroid gland or thyroid glands).af.

10. 7 or 8 or 9

11. (hypophysis thyroid system or thyroid cell or thyroid follicle or thyroid lobe or thyroid parenchyma or thyroid function or euthyroidism).af.

12. 10 or 11

13. 1 or 2 or 3 or 4 or 5 or 6 or 12

14. triclosan/

15. 2' hydroxy 2,4,4' trichlorodiphenyl ether.mp. or triclosan/

16. 2,4,4' trichloro 2' hydroxydiphenyl ether.mp. or triclosan/

17. irgasan dp 300.mp. or triclosan/

18. irgasan dp300.mp. or triclosan/

19. exp methyltriclosan/

20. Irgasan-DP300.mp. or triclosan/

21. (triclosan or 2' hydroxy 2,4,4' trichlorodiphenyl ether or 2,4,4' trichloro 2' hydroxydiphenyl ether or irgasan dp 300 or irgasan dp300 or triclosan methyl or Irgasan-DP300).af.

22. 14 or 15 or 16 or 17 or 18 or 19 or 20 or 21

23. ch 3565.mp. or triclosan/

24. cloxifenol.mp. or triclosan/

25. dp 300.mp. or triclosan/

26. dp300.mp. or triclosan/

27. (ch 3565 or cloxifenol or dp 300 or dp300).af.

28. 22 or 23 or 24 or 25 or 26 or 27

29. 13 and 28

# Risk of bias, observational studies, The Joanna Briggs Institute Critical Appraisal tools

| **Study** | Were the criteria for inclusion in the sample clearly defined? | Were the study subjects and the setting described in detail? | Was the exposure measured in a valid and reliable way? | Were objective, standard criteria used for measurement of the condition? | Were confounding factors identified? | Were strategies to deal with confounding factors stated? | Were the outcomes measured in a valid and reliable way? | Was appropriate statistical analysis used? |
| --- | --- | --- | --- | --- | --- | --- | --- | --- |
| **Almstrup et al. 2020** | Y | Y | Y | Y | Y | Y | Y | Y |
| **Guo et al. 2020** | Y | Y | Y | Y | Y | Y | Y | Y |
| **Ha et al. 2019** | Y | Y | Y | Y | Y | Y | Y | Y |
| **Derakhshan et al. 2019** | Y | Y | Y | Y | Y | Y | Y | Y |
| **Aker et al. 2019** | Y | Y | Y | Y | Y | Y | Y | Y |
| **Skarha et al. 2019** | Y | Y | Y | Y | Y | Y | Y | Y |
| **Berger et al. 2018** | Y | Y | Y | Y | Y | Y | Y | Y |
| **Aker et al. 2018** | Y | Y | Y | Y | Y | Y | Y | Y |
| **Braun et al. 2018** | Y | Y | Y | Y | Y | Y | Y | Y |
| **Wang et al. 2017** | Y | Y | Y | Y | Y | Y | Y | Y |
| **Aker et al. 2016** | Y | Y | Y | Y | Y | Y | Y | Y |
| **Geens et al. 2015** | U (not consecutively includeded) | Y | Y | Y | Y | Y | Y | Y |
| **Koeppe et al. 2013** | Y | Y | Y | Y | Y | Y | Y | Y |

Y: Yes, N: No, U: Unclear, NA: Not applicable

# Risk of bias, interventional studies

| **Study** | **Selection bias** | **Performance bias** | **Detection bias** | **Attrition bias** | **Reporting bias** | **Other biases** |
| --- | --- | --- | --- | --- | --- | --- |
| **Ley et al. 2017** | L | H – no blinding | L | U – lack of information of incomplete outcome data | U – no protocol | - |
| **Palmer et al. 2011** | L | L | L | L | L | - |
| **Allmyr et al. 2009** | NA | H – no blinding | L | L | U – no protocol | - |
| **Poole et al. 2016** | U – no description of randomization method. | L | L | U – lack of handling/information of incomplete outcome data regarding T4. | U – lack of data on T3 in the article in contrast to what the protocol intends. | - |

L: Low risk of bias, H: High risk of bias, U: Unclear risk of bias, NA: Not applicable
